# Dendritic Morphology Affects the Velocity and Amplitude of Back-propagating Action Potentials

**DOI:** 10.1007/s12264-022-00931-9

**Published:** 2022-08-19

**Authors:** Wu Tian, Luxin Peng, Mengdi Zhao, Louis Tao, Peng Zou, Yan Zhang

**Affiliations:** 1 State Key Laboratory of Membrane Biology, College of Life Sciences, Peking University, Beijing, 100871 China; 2 College of Chemistry and Molecular Engineering, Synthetic and Functional Biomolecules Center, Beijing National Laboratory for Molecular Sciences, Key Laboratory of Bioorganic Chemistry and Molecular Engineering of Ministry of Education, Peking University, Beijing, 100871 China; 3 Center for Quantitative Biology, Academy for Advanced Interdisciplinary Studies, Peking University, Beijing, 100871 China; 4 PKU-IDG/McGovern Institute for Brain Research, Peking University, Beijing, 100871 China; 5 Center for Bioinformatics, National Laboratory of Protein Engineering and Plant Genetic Engineering, School of Life Sciences, Peking University, Beijing, 100871 China; 6 Peking-Tsinghua Center for Life Sciences, Peking University, Beijing, 100871 China; 7 Chinese Institute for Brain Research (CIBR), Beijing, 102206 China; 8 Beijing Academy of Artificial Intelligence, Beijing, 100084 China

**Keywords:** Dendrite, Action potential, Back-propagation, Synaptic integration

## Abstract

The back-propagating action potential (bpAP) is crucial for neuronal signal integration and synaptic plasticity in dendritic trees. Its properties (velocity and amplitude) can be affected by dendritic morphology. Due to limited spatial resolution, it has been difficult to explore the specific propagation process of bpAPs along dendrites and examine the influence of dendritic morphology, such as the dendrite diameter and branching pattern, using patch-clamp recording. By taking advantage of Optopatch, an all-optical electrophysiological method, we made detailed recordings of the real-time propagation of bpAPs in dendritic trees. We found that the velocity of bpAPs was not uniform in a single dendrite, and the bpAP velocity differed among distinct dendrites of the same neuron. The velocity of a bpAP was positively correlated with the diameter of the dendrite on which it propagated. In addition, when bpAPs passed through a dendritic branch point, their velocity decreased significantly. Similar to velocity, the amplitude of bpAPs was also positively correlated with dendritic diameter, and the attenuation patterns of bpAPs differed among different dendrites. Simulation results from neuron models with different dendritic morphology corresponded well with the experimental results. These findings indicate that the dendritic diameter and branching pattern significantly influence the properties of bpAPs. The diversity among the bpAPs recorded in different neurons was mainly due to differences in dendritic morphology. These results may inspire the construction of neuronal models to predict the propagation of bpAPs in dendrites with enormous variation in morphology, to further illuminate the role of bpAPs in neuronal communication.

**Supplementary Information:**

The online version contains supplementary material available at 10.1007/s12264-022-00931-9.

## Introduction

In most mammalian central nervous system neurons, the action potential (AP) is initiated at the proximal axon region and propagates forward and backward [1]. The part of the AP invading the soma and the dendritic tree is called a back-propagating AP (bpAP), and active bpAPs have been reported in many types of neurons, both *in vitro* and *in vivo* [2–10].

The most notable role of bpAPs is their participation in spike timing-dependent plasticity (STDP), including long-term potentiation (LTP) and long-term depression (LTD). The induction of STDP requires pre- and postsynaptic activity to occur in a precise temporal order within a window of only tens of milliseconds [11–13], and the invasion of distal dendrites bpAPs is an important source of depolarization [14]. The amplitude of a bpAP may determine the strength of subsequent postsynaptic depolarization and affect the induction of plasticity. The velocity and frequency of bpAPs influence the timing of postsynaptic activity and the chance of potentiation. Therefore, the properties of bpAPs have a critical influence on the integration of synaptic input and the induction of synaptic plasticity [15, 16]. Understanding the ways in which different factors determine the extent of bpAP propagation in dendrites is critical for studies of the functions of bpAPs.

The properties of bpAPs in different types of neurons vary widely. The average velocity of bpAPs in granule cell dendrites is 150 μm/ms [17], which is slower than their velocity in pyramidal cell apical dendrites (500 μm/ms) and basal dendrites (200 μm/ms) [18–20]. The marked differences among bpAPs on the dendrites of different neuronal types inspired us to investigate the effect of morphology on bpAPs, with a focus on how the diameter and branch pattern influence bpAP velocity and amplitude.

Most previous studies of bpAPs have used direct dendritic recordings, but their low spatial resolution is a serious drawback. It is extraordinarily difficult to figure out how bpAPs propagate and change between two recording sites using traditional electrophysiological methods, and the reported velocity and amplitude of bpAPs are values obtained from dendrites with lengths of hundreds of micrometers. Without an exhaustive recording and description of bpAPs, it is difficult to reveal how factors influence them and further understand their functions. To overcome this limitation, recent studies have applied optical recording *via* genetically-encoded voltage indicators (GEVIs) or genetically-encoded Ca^2+^ indicators [21, 22]. Here we used a previously developed all-optical electrophysiological method using a GEVI [23] to record the membrane voltage in the dendrites of cultured hippocampal neurons. This “Optopatch” method improves spatial resolution to the single-micron level and possesses sub-millisecond temporal resolution. Previous studies have generally examined only one dendrite in detail, but all-optical voltage imaging allowed us to simultaneously record and study bpAPs in several proximal dendrites from one neuron in detail without perturbation. By comparing the morphology of individual dendrites and the corresponding bpAPs, we were able to understand how the bpAP propagates in the dendrite in detail and study the effect of dendritic diameter and branching on bpAP velocity and amplitude more quantitatively.

We also used computational neuron modeling to simulate the backpropagation of APs in dendrites with various morphological features, to obtain more precise correlations between morphology and bpAP properties. Our models and experimental data together revealed that the varying properties of bpAPs in dendrites are primarily determined by dendritic morphological features. These results have important implications for our understanding of bpAP modulation, and they provide a foundation for the construction of a universal neuron model capable of reproducing the propagation of a bpAP on a dendrite with any given morphology.

## Materials and Methods

### Primary Neuron Culture

Primary neurons were cultured from postnatal day 0 C57BL/6J mice following the rules and regulations of the Peking University Animal Care and Use Committee as described previously [24]. Fresh hippocampal tissue was dissected from the brain and digested with 0.25% trypsin (Gibco, NY, USA) for 15 min in an incubator at 37 °C, followed by inactivation with an equal volume of DMEM/F12 medium (Gibco) with 10% fetal bovine serum (FBS) (Gibco). Next, the mixture was lightly triturated using a pipette. After 2 min of precipitation, the supernatant was collected and centrifuged at 200× g for 5 min. The cell sediment was re-suspended in DMEM/F12 medium with 10% FBS and plated on coverslips coated with poly-D-lysine (Sigma, NJ, USA) at a density of 1 × 10^5^ neurons/coverslip in a 24-well culture dish. After half an hour, the cells were maintained with Neurobasal medium (Gibco) containing 1% penicillin-streptomycin (Gibco), 2% B27 (Gibco), and 1% GlutaMAX (Gibco). Half of the culture medium was replaced with fresh medium every 2 days.

### Ca^2+^-Phosphate Transfection

Neurons were transfected on day 8 *in vitro* (DIV8) with Optopatch2 plasmids *via* the Ca^2+^ phosphate transfection method. FCK-Optopatch2 plasmid was a gift from Prof. Adam. E. Cohen, Harvard University. Generally, 2 μg of plasmid diluted in CaCl_2_/water solution was mixed with an equal volume of 2× HEPES balanced salt solution (HBS) (Clontech, Shiga, Japan) followed by gentle vortexing. The DNA-Ca^2+^-phosphate complex was added dropwise to neurons maintained in fresh Neurobasal medium. The neurons were incubated at 37 °C for 1 h and then washed twice with 1× HBS (pH 6.8) to completely clear the precipitate. Finally, cells were maintained in their original growth medium in an incubator until DIV12 for imaging as described below.

### Immunocytochemistry

The neurons were fixed in 4% paraformaldehyde for 15 min, permeabilized for 10 min with 0.2% Triton X-100 in phosphate-buffered saline (PBS), and blocked with blocking buffer (5% bovine serum albumin and 0.2% Tween 20 in PBS). The neurons were incubated at 4 °C overnight with primary antibodies diluted in blocking buffer to the appropriate concentration. After washing with PBS, the neurons were incubated with secondary antibodies for 1 h at room temperature. Finally, the coverslips with neurons were mounted on slides with Fluoromount-G (SouthernBiotech, AL, USA). The following antibody dilutions were used: anti-PROX1 (1:500, Abcam, Cambridge, UK), anti-GAD65 (1:500, Abcam), anti-GFP (1:2000, Abcam), and all secondary antibodies (1:500, Invitrogen, NY, USA).

### Imaging Apparatus

Fluorescent images were collected on a spinning disk confocal microscope (Andor Dragonfly, Oxford Instruments, Oxford, UK) equipped with a 40× 1.3 NA oil immersion objective lens, 4 laser lines (405, 488, 561, and 637 nm), and electron-multiplying charge-coupled device (EMCCD) or scientific complementary metal-oxide-semiconductor (sCMOS) cameras (Andor).

All-optical electrophysiological experiments were conducted on an inverted fluorescence microscope (Nikon-TiE, Nikon, Tokyo, Japan) equipped with a 40× 1.3 NA oil immersion objective lens (Nikon CFI Plan Fluor), 6 laser lines (Coherent OBIS, 405, 488, 532, 594, 561, and 639 nm), and an sCMOS camera (Hamamatsu ORCA-Flash 4.0 v2, Hamamatsu, Shizuoka, Japan). The microscope, lasers, cameras, and high-precision stage (Marzhauser Wetzlar, Hessian, German) were controlled with customized software programmed in LabVIEW (National Instruments, 15.0 version, TX, USA). Most fluorescent proteins were imaged at illumination intensities of 1–6 W/cm^2^, while QuasAr2 was imaged at 800–1000 W/cm^2^.

### Fluorescent Imaging of Neurons

The fixed neurons were imaged on a Dragonfly confocal microscope (Andor). Images were acquired at 1 × 1 camera binning with an exposure time of 100–1000 ms in *Z*-stack mode with a 0.5-μm step length. In ImageJ/Fiji [25], each image stack was *Z*-projected with maximum intensity to one image, and new images in several channels were adjusted and merged.

### Whole-cell Patch-clamp with Simultaneous Voltage Imaging

Before imaging and electrophysiology, neurons on coverslips were placed in a glass-bottomed dish filled with customized high-glucose Tyrode’s solution containing (in mmol/L) 125 NaCl, 2.5 KCl, 3 CaCl_2_, 1 MgCl_2_, 10 HEPES, and 30 glucose (pH 7.3, adjusted to 305–310 mOsm/kg with sucrose). The synaptic blockers NBQX (10 μmol/L), AP-V (25 μmol/L), and gabazine (20 μmol/L, all from Sigma) were added to the buffer for measurements of single-cell electrophysiology. All experiments were performed at room temperature (~22 °C).

Borosilicate glass electrodes (WPI, FL, USA) were pulled to a tip resistance of 2.5–5 MΩ. The electrodes were filled with internal solution containing (in mmol/L) 125 potassium gluconate, 8 NaCl, 0.6 MgCl_2_, 0.1 CaCl_2_, 1 EGTA, 10 HEPES, 4 Mg-ATP, 0.4 Na_2_-GTP (adjusted to pH 7.3 with KOH and 290–300 mOsm/kg with 1 mol/L sucrose). Neurons were clamped in the whole-cell current-clamp mode, and the membrane voltage signal recorded from the patch amplifier (Axopatch 200B, Molecular Devices, CA, USA) was filtered with an internal 5-kHz Bessel filter and digitized at 9681.48 Hz with a National Instruments (TX, USA) PCIe-6353 data acquisition board. During the current stimulation, simultaneous voltage images were acquired at a 20× down-sampling rate (484.07 Hz, 2.0658 ms/frame).

### All-optical Electrophysiology

Imaging experiments were conducted in the same external solution. We coupled an sCMOS camera and a 488-nm laser, which were controlled by LabVIEW software to achieve simultaneous optogenetic stimulation and imaging. The signal rate of laser control and patch-clamp recording was 4643.60 Hz, with 10× up-sampling to camera acquisition (464.36 Hz, 2.1535 ms/frame). We used a 488-nm laser pulse (0.05–0.20 W/cm^2^) with a duration of 1.93 ms to illuminate neurons at a rate of 5 Hz, which stimulated neurons to generate 50 APs in 10 s. Meanwhile, we used a 639-nm laser to excite QuasAr2. The two beams were combined and projected onto the neuron through the objective lens. Fluorescent signals were collected by the sCMOS camera under rolling-shutter mode with 2 × 2 binning.

### Quantitative Analysis of Video Data

The relative fluorescence change (Δ*F*/*F*) was used to derive the AP signal. To obtain a spike-triggered average movie, the signal of every pixel was averaged over all simulated APs (by peak-finding and alignment) to boost the signal-to-noise ratio (SNR) for the following interpolation and calculation. The camera bias (intensity of 400 in the 2 × 2 binning mode) was subtracted from these average intensities to give the final values. Then we applied photobleaching correction of the fluorescent signal by dividing the raw signal with a reference baseline, which was constructed from processing the sliding minimum filter on the fluorescent signals.

To quantify the bpAP propagation, we processed the video data by a self-developed algorithm with MATLAB (version R2020a, MA, USA). A flowchart of the algorithm is shown in Fig. S1A. First, we drew the center line of the dendrite of interest (Fig. S1B). Next, a 5 pixels × 5 pixels region centered by each pixel on the center line was set as the whole region of interest (ROI), and the intensity of the whole ROI was smoothed to obtain the photobleaching baseline. The final value we used was the intensity divided by the photobleaching value (Fig. S1C). After that, we found the peaks (local maxima) of periodic APs (Fig. S1D upper) and averaged them to obtain the mean AP trace. To increase the time resolution, we interpolated the mean AP trace with a cubic spline to obtain a green AP trace, which was used as a kernel to revise the APs in each segment. The cubic spline interpolation increased the time resolution of the data from 2 ms to 0.002 ms (Fig. S1D lower).

Next, we calculated the peak time of the AP at each pixel on the center line. The peak time was averaged within an 11-pixel-long window sliding on the center line, which contained 5 pixels before and after the central pixel (Fig. S1E). We first averaged the signals of pixels in this window to obtain the signal on the red point (blue signal) that was the central point. Then, we interpolated the signal of the red point with the cubic spline (red signal) and convolved the splined signal with the kernel (Fig. S1D) to calculate the correlation coefficient (yellow line in Fig. S1E). Then we replaced the splined signal according to the correlation coefficient to obtain the corrected signal (purple signal). After that, we used this corrected, high-precision signal to find the relative peak arrival time of the red point’s AP. Finally, we calculated the bpAP propagation velocity in the dendrite (Fig. S1F). The change of the relative fluorescence signal after interpolation was used to calculate the relative amplitude of the bpAP.

### Modeling a Neuron

We created a detailed multi-compartment model of a neuron to explore the characteristics of backpropagating signals. The model included simplified morphology, ion channel distributions and densities, channel kinetics, and passive properties. The neuronal morphology was modeled with simple stick models consisting of a cylindrical soma (1 μm in length × 20 μm in diameter) attached to 8 dendrites with different diameters (when simulating how diameter influences velocity) or to a binary tree (when simulating how the branch point influences velocity). We stimulated the model neuron with an AP-shaped voltage stimulus injected directly into the soma, after which the signal backpropagated to the dendrites. The simulation was implemented within the simulation software NEURON (version 7.6.5 running on Microsoft Windows 10). The integration time steps were fixed at 0.001 ms. The length of all segments was 1 μm. The full model is available on ModelDB (https://senselab.med.yale.edu/modeldb/ShowModel?model=267417, access code: DendriteSimCode). Analysis of the simulation data was performed with MATLAB (version R2020a).

The parameters used in the simulations are listed in Table 1. Various passive parameter values were explored within physiologically relevant ranges taken from the literature [26–30]. The specific values of the passive parameters were chosen in order to match the experimentally measured input resistance and membrane time constant in mouse hippocampal neurons.

**Table 1:** Parameters used in the simulations

Model parameter	
Passive properties	Default
Specific membrane capacitance (*C*_m_)	0.5 $\mathrm{\mu F}/{\mathrm{cm}}^{2}$
Specific membrane resistance (*R*_m_)	20,000 $\Omega {\mathrm{cm}}^{2}$
Intracellular resistivity (*R*_a_)	300 $\Omega \mathrm{cm}$
Leak reversal potential (*E*_L_)	−65 $\mathrm{mV}$
Active properties	
${\overline{G} }_{\mathrm{Na}}$(maximum Na^+^ conductance)	100 $\mathrm{pS}/{\mathrm{\mu m}}^{2}$
${\overline{G} }_{\mathrm{K}}$(maximum K^+^ conductance)	100 $\mathrm{pS}/{\mathrm{\mu m}}^{2}$

With regard to active properties, the gating kinetics of voltage-dependent Na^+^ and K^+^ channels were modeled after the standard Hodgkin-Huxley model [31, 32]. The kinetics and parameters of the ion channels are listed in Table 2. We adjusted the ion channel densities according to experimentally obtained densities from the literature [26, 29, 31].

**Table 2:** Kinetics of voltage-dependent Na^+^ and K^+^ channels

Channel type	Gating particle	Dynamics
Na^+^ channel		${I}_{\mathrm{Na}}={G}_{\mathrm{Na}}({V}_{\mathrm{m}}-{E}_{\mathrm{Na}})$
		$${G}_{\mathrm{Na}}={\tau }_{\mathrm{adj}}{\overline{G} }_{\mathrm{Na}}{m}^{3}h$$
		$${\tau }_{\mathrm{adj}}={2.3}^{\frac{T-23}{10}}$$
	*m*	$\dot{m}=\frac{{m}_{\infty }-m}{{\tau }_{m}}$
		$$ \alpha _{m} = 1.638 \cdot {\text{exp}}(\frac{{ - V_{{\text{m}}} - 35}}{9}) $$
		$${\beta }_{m}=1.116\cdot \mathrm{exp}(\frac{{V}_{\mathrm{m}}+35}{9})$$
		$${m}_{\infty }=\frac{{\alpha }_{m}}{{\alpha }_{m}+{\beta }_{m}}$$
		$${\tau }_{m}=\frac{1}{{\tau }_{\mathrm{adj}}({\alpha }_{m}+{\beta }_{m})}$$
	*h*	$\dot{h}=\frac{{h}_{\infty }-h}{{\tau }_{h}}$
		$${\alpha }_{h}=0.120\cdot \mathrm{exp}(\frac{-{V}_{\mathrm{m}}-50}{5})$$
		$${\beta }_{h}=0.046\cdot \mathrm{exp}(\frac{{V}_{\mathrm{m}}+75}{9})$$
		$${h}_{\infty }=\frac{1}{1+\mathrm{exp}(\frac{{V}_{\mathrm{m}}+65}{6.2})}$$
		$${\tau }_{h}=\frac{1}{{\tau }_{\mathrm{adj}}({\alpha }_{h}+{\beta }_{h})}$$
K^+^ channel		${I}_{\mathrm{K}}={G}_{\mathrm{K}}({V}_{\mathrm{m}}-{E}_{\mathrm{K}})$
		$${G}_{\mathrm{K}}={\tau }_{\mathrm{adj}}{\overline{G} }_{\mathrm{K}}n$$
	*n*	$\dot{n}=\frac{{n}_{\infty }-n}{{\tau }_{n}}$
		$${\alpha }_{n}=0.180\cdot \mathrm{exp}(\frac{{-V}_{\mathrm{m}}+25}{9})$$
		$${\beta }_{n}=0.018\cdot \mathrm{exp}(\frac{{V}_{\mathrm{m}}-25}{9})$$
		$${n}_{\infty }=\frac{{\alpha }_{n}}{{\alpha }_{n}+{\beta }_{n}}$$
		$${\tau }_{n}=\frac{1}{{\tau }_{\mathrm{adj}}({\alpha }_{n}+{\beta }_{n})}$$

*I*, current; *G*, channel conductance; ${\overline{G} }$, the maximum channel conductance; *V*_m_, membrane potential; *E*, reversal potential of the ion channel; ${\tau }_{\mathrm{adj}}$, temperature coefficient; *T*, temperature. *x* is the gating parameter ($x=m, h,n$), $\dot{x}$ is the derivative of *x* with respect to time, ${\alpha }_{x}$ is the opening rate of gate, ${\beta }_{x}$ is the closing rate of gate, ${x}_{\infty }$ is the limiting value that *x* approaches, with the time constant ${\tau }_{x}$.

According to Rall’s study [33], a total of *k* dendrites at the same level with arbitrary diameters ${r}_{k}$ are equivalent to one dendrite of diameter ${r}_{0}$ as follows:

$${r}_{0}^\frac{3}{2} = \sum_{k}{{r}_{k}}^\frac{3}{2}$$

In simulations of multi-level binary trees, the sum of the diameters of two daughter dendritic cables after the branching point is equivalent to that of the mother dendritic cable, according to this formula.

### Statistical Analysis

We defined the primary dendrite as the dendrite that arose directly from the soma with a length of at least 5 μm, and the secondary dendrites were defined as the dendrites branching from the primary dendrite with a length of at least 5 μm. Diameter and soma area measurements were made on image stacks using Fiji/ImageJ. Data were analyzed using Prism 7.0 (GraphPad, CA, USA). All values are given as the mean ± SEM unless otherwise noted. Two-tailed Student’s *t*-test and one-way ANOVA with Dunnett’s *post hoc* test were used for data analysis. Reported *P-*values indicate statistical significance as follows: *P <0.05, **P <0.01, ***P <0.001, and ****P <0.0001.

## Results

### Sub-millisecond-level All-optical Electrophysiological Techniques Resolve Backpropagating Action Potentials Simultaneously in Several Dendrites

**Fig. 1:**
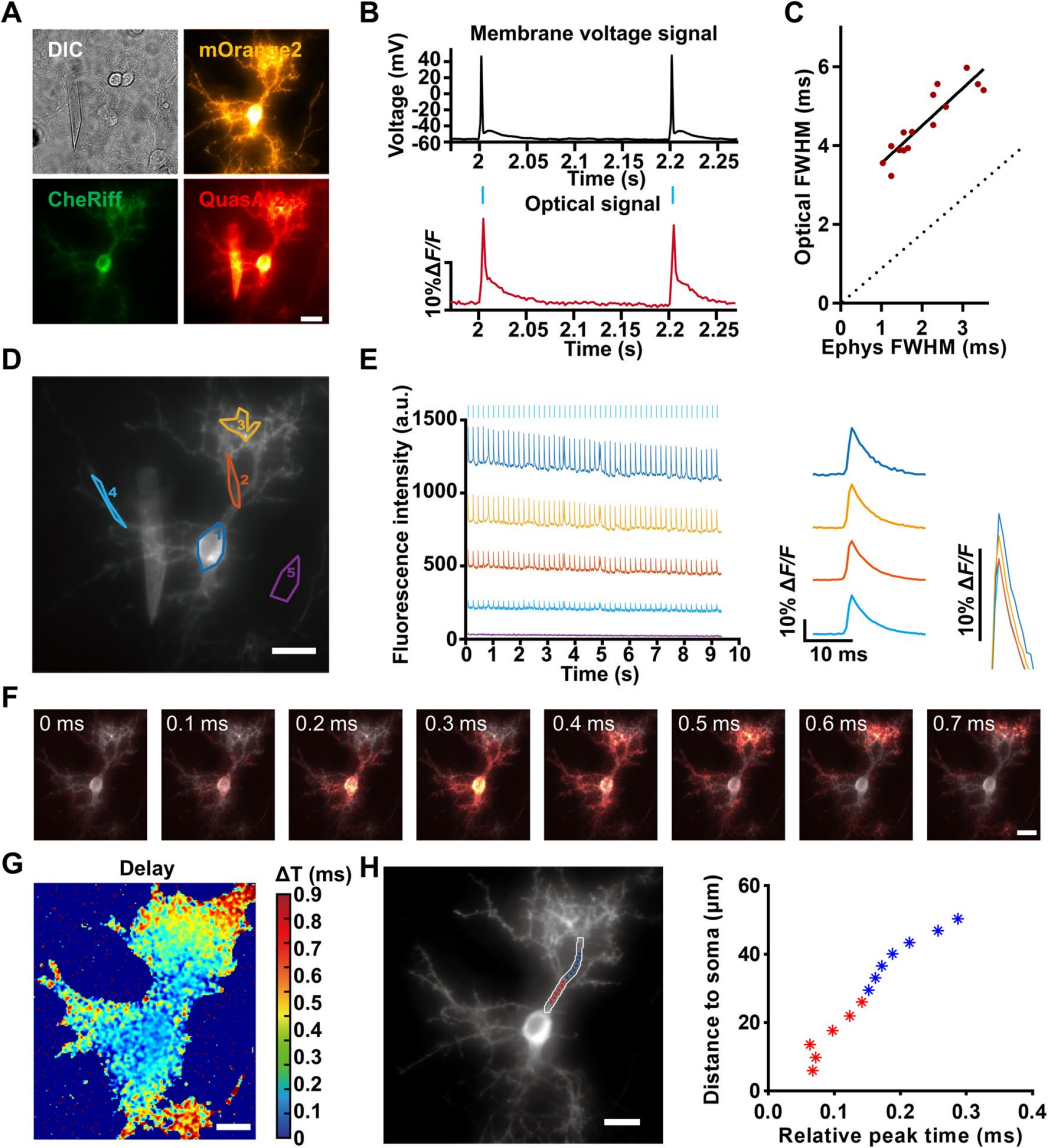
Studying backpropagating action potentials in dendrites *via* all-optical electrophysiology. **A** Images of a neuron expressing Optopatch2 elements. Scale bar, 10 µm. **B** Simultaneous fluorescence and patch-clamp recordings from one neuron. Blue bar, illumination; red trace, whole-cell single-trial fluorescence; black trace, patch-clamp recording. **C** FWHM comparison of AP waveforms from electrical and optical signals. Ephys FWHM, FWHM of the AP signal waveform recorded by the patch clamp. **D** Several regions in the whole view of one sample neuron, including the soma, primary dendrites, secondary dendrites, and background. Scale bar, 10 µm. **E** Fluorescence signal in several regions marked in **C** during simulation. Left, blue marks at the top of the left panel indicate blue light stimulation. Right, average Δ*F*/*F* of QuasAr2 fluorescence in several regions (colors of signals represent regions marked by the same colors in **D**). **F** Frames from a propagation video showing the delay of the bpAP on the dendrite relative to the soma. Scale bar, 10 µm. **G** Peak time delay heatmap of a bpAP in the neuron shown in **E**. Scale bar, 10 µm. **H** Image and plot of peak time *versus* distance from the soma of a bpAP propagating along one dendrite (red, propagation on the primary dendrite; blue, propagation on the secondary dendrite). Scale bar, 10 µm.

Optical recording using GEVI can, in principle, overcome many of the technical limits encountered in traditional electrophysiological recordings, providing a way to record APs at subcellular regions or at multiple sites with maximum fidelity and millisecond response times. The Optopatch2 construct we used contained CheRiff, a channelrhodopsin, as the AP trigger, as well as QuasAr2, a near-infrared voltage indicator. These two proteins were joined by a self-cleaving 2A peptide sequence to ensure that they were expressed at similar levels. After expression in cultured mouse hippocampal neurons, both proteins showed extensive membrane trafficking in all regions of the neuron, including the soma, axon, proximal dendrites, and distal dendrites (Fig. 1A).

To test whether the fluorescent kinetics of QuasAr2 faithfully reflect the AP waveform, the electrical signal through a patch-clamp and the optical signal after evocation of APs *via* optogenetic stimulation were simultaneously recorded and compared. Imaging data were processed as previously reported [23]. QuasAr2 showed high fluorescent sensitivity and a fast response speed to potential changes, and the synchronization of the traces of the electrical and optical signals was good using the optical stimulation approach (Fig. 1B). We also compared the full width at half maximum (FWHM) of AP waveforms under these two recording methods. The results showed that the FWHM of AP waveforms represented by the optical signal was larger than that of the electrical signal, which was acceptable using a voltage indicator. Moreover, there was still a positive correlation between the FWHM of AP waveforms from the two recording methods, indicating a fast response speed of QuasAr2 and high fidelity of AP waveforms (Fig. 1C).

Next, we used this all-optical electrophysiological method to record and reveal the propagation of bpAPs in the dendritic tree. Subsequent images were recorded at a 484-Hz frame rate for 10 s. Under optical stimulation, membrane voltage dynamics were simultaneously recorded at the soma and dendrites of a single neuron in the field of view. bpAPs were observed in the different regions of the dendrite with perfect synchronization to the somatic APs, but with an attenuated peak (Fig. 1D, E).

Spike-triggered average movies from raw voltage imaging data were generated to improve the SNR and for subsequent analysis. An interpolation algorithm was used to analyze the AP timing with sub-millisecond temporal resolution and pixel-level spatial resolution (see Materials and Methods). The AP propagation heatmap showed that APs occurred initially at the soma and were then conducted into the dendrites (Fig. 1F, G, and Video S1).

We obtained information about APs on the whole dendrite within the field of view. This improved the precision and spatial resolution of bpAP recording and made it possible to analyze the bpAP propagation process in detail. By quantifying this process, we were able to obtain the average bpAP velocity at any given dendrite segment, which could consist of the whole primary dendrite or only a part of it. The results clearly showed that the bpAP propagation velocity in dendrites fluctuated (Fig. 1H).

### bpAP Properties Are Mainly Determined by Dendritic Tree Morphology

**Fig. 2:**
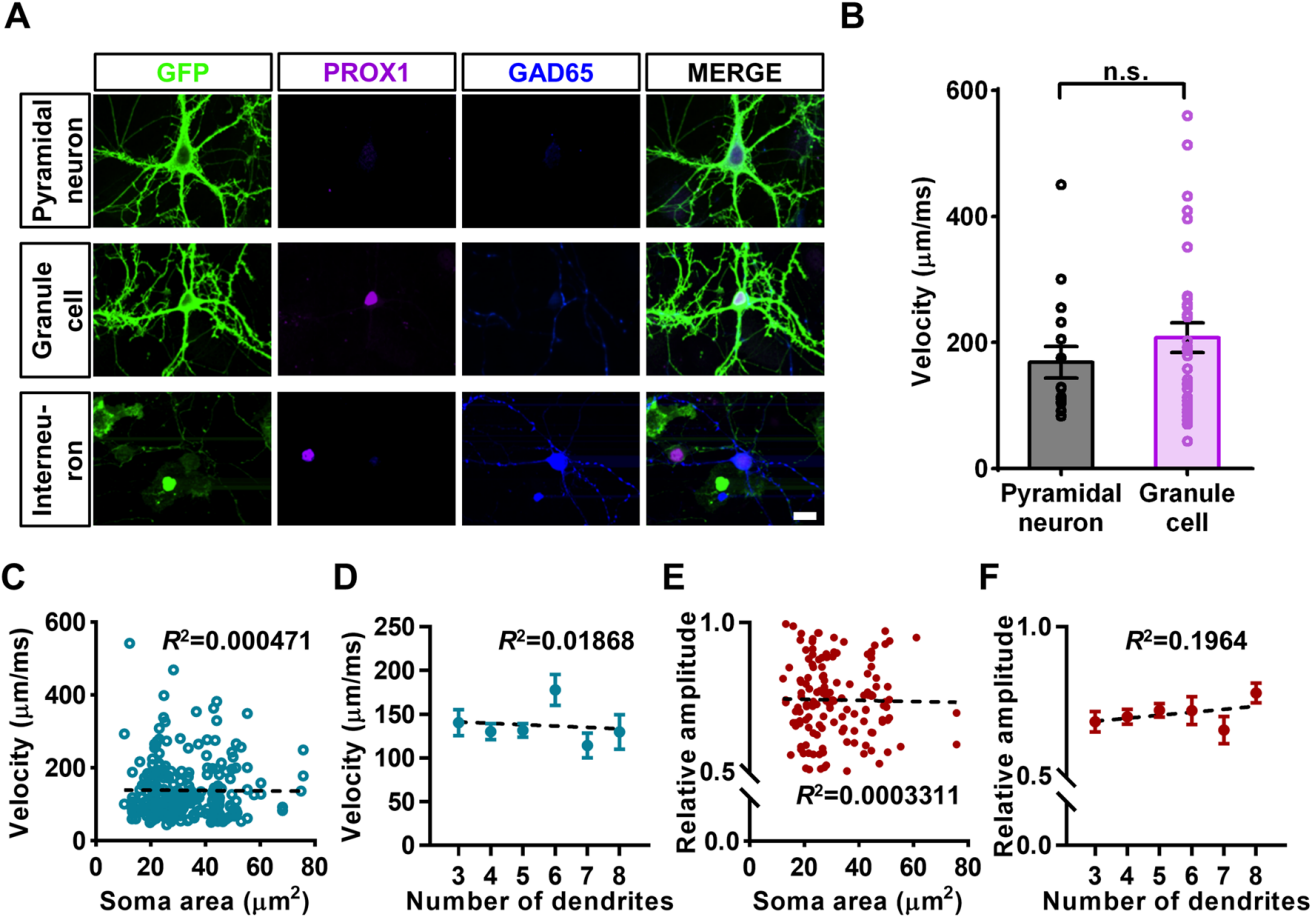
The soma area and the number of primary dendrites are not correlated with bpAP variation among neurons. **A** Immunostaining of different types of neurons. GFP indicates neurons expressing Optopatch2. Scale bar, 10 µm. **B** Velocity of bpAPs in pyramidal neurons (*n* = 26) and granule cells (*n* = 52). *P* = 0.8211; n.s., no significant difference, unpaired *t*-test. Error bars, SEM. **C** Plot of the correlation between bpAP velocity on primary dendrites and soma area (*n* = 252). **D** Plot of the correlation between bpAP velocity and the number of primary dendrites. PD, primary dendrite. Column 3: *n* = 46; column 4: *n* = 49; column 5: *n* = 75; column 6: *n* = 31; column 7: *n* = 20; column 8: *n* = 17; all *P* >0.05, one-way ANOVA. Error bars, SEM. **E** Plot of the correlation between the relative amplitude of bpAPs and soma area. *n* = 143. **F** Plot of the correlation between the relative amplitude of bpAPs and the number of primary dendrites. Column 3: *n* = 28; column 4: *n* = 38; column 5: *n* = 51; column 6: *n* = 15; column 7: *n* = 12; column 8: *n* = 11; all *P* >0.05, one-way ANOVA. Error bars, SEM.

Previous studies have shown that the propagation velocity of bpAPs differs markedly between pyramidal neurons and granule cells in brain slices, as well as between the apical and basal dendrites of a single pyramidal neuron. To separate recorded neurons into different cell types, we fixed and stained the neurons with several cell markers after acquiring voltage imaging data. Anti-PROX1 antibodies were used to label granule cells, while anti-GAD65 antibodies were used to indicate inhibitory neurons. As neurons transfected with Optopatch2 expressed GFP and mOrange2 at the same time, it was nearly impossible to use another cell marker with an unappropriated fluorescence channel. Therefore, we considered neurons lacking both the PROX1 and GAD65 signals to be pyramidal neurons (Fig. 2A).

No inhibitory neurons were recorded because the promoter of the plasmid was CamKIIa. After classifying the analyzed neurons into different types, we found that there was no significant difference in the average velocities of bpAPs in pyramidal neurons (190 μm/ms) and granule cells (202 μm/ms) (Fig. 2B).

The lack of significant differences between the average velocities of bpAPs in different neuron types might have been a result of differences induced by culturing neurons on coverslips rather than obtaining them from brain slices. The dendritic morphology varied markedly between cultured neurons and neurons in brain slices. The 2D culture conditions might hide the structural and morphological distinctions between different cell types and unique dendrites. These results indicated that the bpAP propagation processes in neurons with similar dendritic tree morphology were identical. This finding inspired us to investigate the basic principles underlying the effect of dendritic morphology on bpAP propagation that could apply to all types of neuron.

Previous studies considered dendritic morphology to be the main factor that determines the main extent of bpAPs. Besides dendritic morphology, other morphological features of a neuron, like its volume and the number of primary dendrites, may also influence bpAP propagation. Therefore, we designed experiments to determine whether the volume of the cell body and the number of dendrites influences bpAP propagation. In cultured neurons, the soma area was used as a substitute for its volume. We found no direct correlation between the soma area or the number of primary dendrites and bpAP velocity (Fig. 2C, D). Since the fluorescence intensity of QuasAr2 varied among the tested neurons, we posited that the AP amplitudes at the somas of different neurons were nearly equal, and we used the ratio of relative fluorescence change from the dendrites to the soma to represent the relative bpAP amplitude. In addition, we found no correlation between the relative bpAP amplitude and the soma area or the number of primary dendrites (Fig. 2E, F). This finding indicated that the volume of the cell body and the number of primary dendrites have little influence on bpAP propagation.

### bpAP Velocity is Positively Correlated with Dendritic Diameter in Individual Neurons

**Fig. 3:**
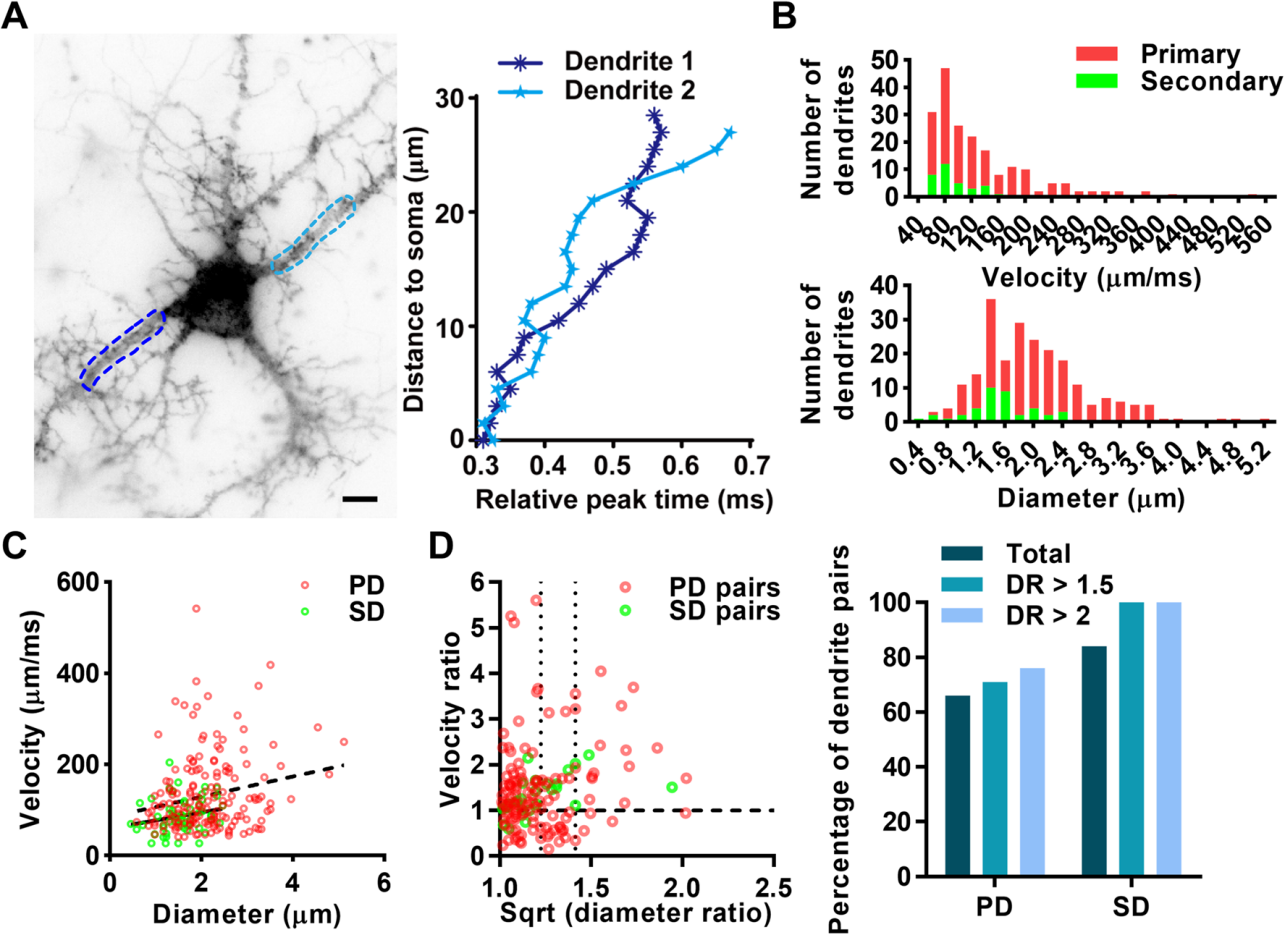
The velocity of bpAPs is positively correlated with dendritic diameter in a single neuron. **A** Plot of the peak time of bpAPs on two dendrites of one neuron *versus* distance to the soma. The data on the dendrite segment marked by a dotted line are shown in the same color in the right panel. Scale bar, 10 µm. **B** Frequency distribution of the velocity of bpAPs and dendritic diameter on primary (PD; *n* = 182) and secondary dendrites (SD; *n* = 40). **C** Plot of the correlation between bpAP velocity and dendritic diameter (red, primary dendrites, *n* = 179; green, secondary dendrites, *n* = 56). **D** Left, Plot of the correlation between the velocity ratio and the square root (Sqrt) of the dendritic diameter ratio in primary (PD, *n* = 132) and secondary dendrite pairs (SD, *n* = 25). Right, percentage of dendrite pairs showing a positive relationship between bpAP velocity and dendritic diameter in primary and secondary dendrites when the diameter ratio (DR) is in different ranges: total, PD: 67%, SD: 84%; when the diameter ratio >1.5, PD: 71%, SD: 100%; when the diameter ratio >2, PD: 76%, SD: 100%.

The diameter of multi-level dendrites is an important morphological characteristic that varies within a relatively large range. The effects of this variation were reflected in the average velocity, as well as the pattern with which the velocity changed (Fig. 3A). We investigated the correlation between bpAP velocity and dendritic diameter. The average bpAP velocity was 128 ± 6 μm/ms on the primary dendrite and 81 ± 5 μm/ms on the second dendrite (Fig. 3B). These results were similar to those of previous electrophysiological studies. The average primary dendrite diameter was 2.08 ± 0.05 μm, and the average secondary dendrite diameter was 1.51 ± 0.08 μm. We found a trend indicating a positive correlation between bpAP velocity and dendritic diameter (Fig. 3C), although there was no simple and direct relationship. To obtain a more relevant correlation, we divided primary dendrites from the same neuron or secondary dendrites from one primary dendrite into dendrite pairs in which the thinner dendrite was the reference. Then we calculated their velocity ratio and the square root of the diameter ratio based on previous studies of AP propagation simulation [34]. The velocity ratios of 67% of primary dendrite pairs and 85% of secondary dendrite pairs were each >1, indicating that the bpAP velocity on the thicker dendrite was faster (Fig. 3D). This positive correlation was more significant in secondary dendrites, and might result from a higher level of homogeneity between two secondary dendrites branching from a single primary dendrite than that between two primary dendrites from the same neuron. As dendrites are relatively small, two dendrites with a small dendritic ratio may not differ markedly; therefore, we examined dendrite pairs with diameter ratios >1.5 and dendrite pairs with diameter ratios >2. As the diameter ratio increased, the proportion of dendrite pairs with positively correlated bpAP velocities also increased (Fig. 3E). Therefore, for two dendrites from a single neuron, a bpAP is likely to propagate more rapidly on the thicker dendrite. When an AP is initiated at the axon initial segment (AIS) and then backpropagates into dendrites through the cell body, a bpAP on a thick dendrite would be very likely to propagate faster and reach its destination earlier than those on other dendrites.

### bpAP Velocity is Negatively Correlated with the Density of Dendritic Spines

Spines are unique structures in dendrites. Due to the use of synaptic blockers, in our study, dendritic spines did not accept synaptic signal inputs, so the spine could be regarded as the structure that enlarges the volume and membrane area of dendrites to a certain extent. The distributions of spines on the dendrites of the neurons recorded in our experiment were very different, therefore we explored whether the density of spines also affected the velocity of the bpAP. We found that even in one neuron, there were dendrites with similar diameters but quite different spine densities (Fig. S2A). And the velocities of bpAPs on these two dendrites were different as well, not just in the average velocity but also in how the velocity changed. The average velocity of the bpAP on the dendrite with a low density of spines was much higher (Fig. S2B).

Then, we selected dendrite pairs from different neurons with similar dendrite diameters and markedly different spine densities. The diameter ratio of the two selected dendrites was ≤1.1 (taking the thinner dendrite as reference), and the density ratio of spines was ≥1.5 (taking the dendrite with fewer spines as reference). Based on the density of spines on two dendrites, we divided every dendrite pair into “sparse” and “dense” groups and compared the velocities of bpAPs on them. The velocity of bpAPs on the dendrites in the sparse group was significantly higher than that in the dense group (Fig. S2C). In order to further confirm this result, we also selected dendrites with similar diameters but different spine densities from the same neuron, and compared the velocities of bpAPs on them. The result showed that the velocity of bpAPs on dendrites with fewer spines from the same neuron was clearly faster (Fig. S2D).

These results indicated that when the diameter of dendrites was similar, the velocity of the bpAP was negatively correlated with the density of dendritic spines. Therefore, the difference in the density of spines may increase the difference between the dendrites of different neurons, and result in an indirect and significant correlation between the velocity of the bpAP and the square root of the diameter of the dendrite.

### bpAP Amplitude, but Not Attenuation, is Positively Correlated with Dendritic Diameter in Individual Neurons

**Fig. 4:**
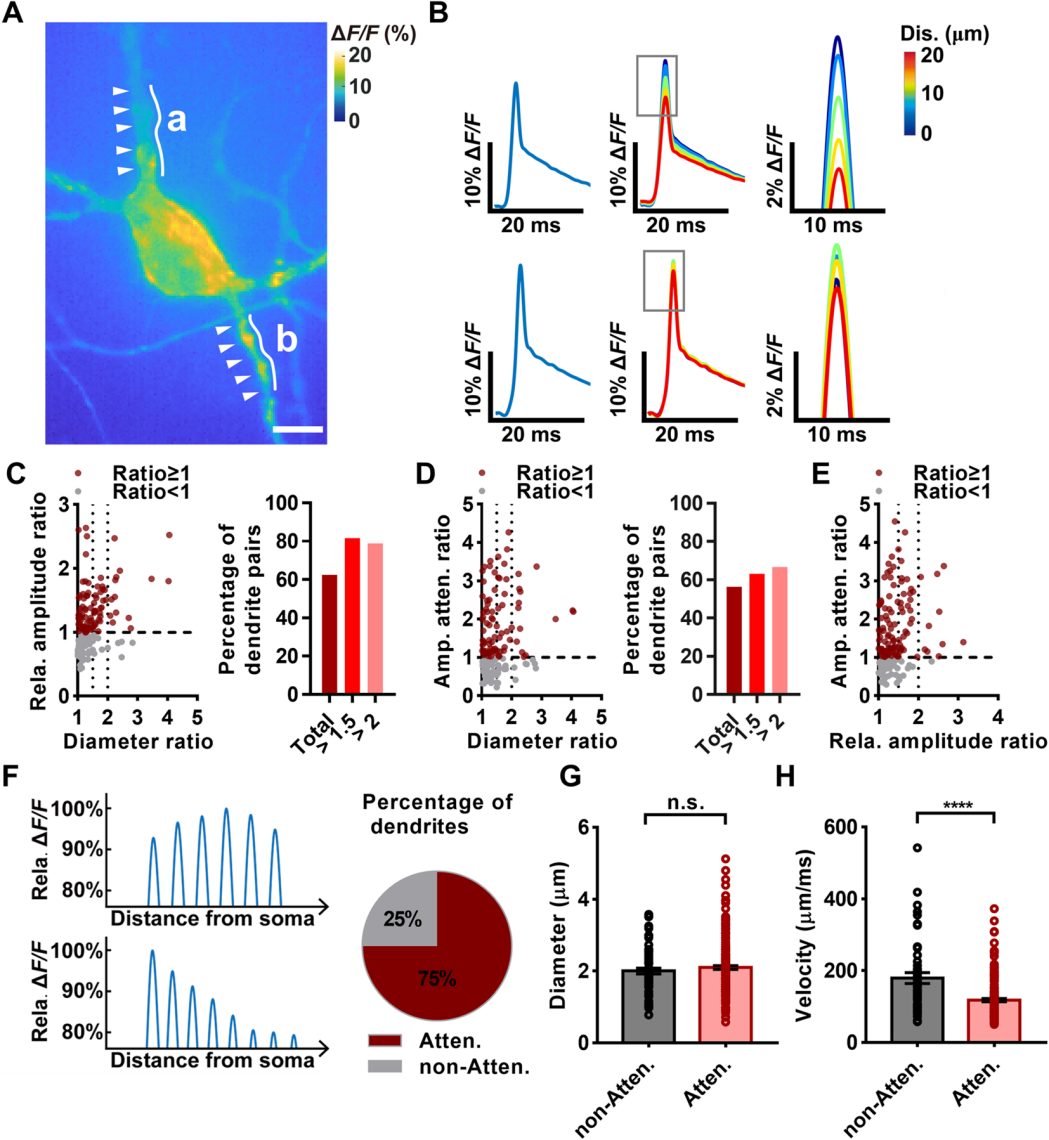
The amplitude and attenuation of bpAPs are correlated with different factors in a single neuron. **A** Representative neuron with different bpAP amplitudes on different dendrites (a and b) (arrowheads, position of bpAP recording). Scale bar, 10 µm. **B** Waveforms of bpAPs on dendrites of **A**. Left, the average waveform of bpAPs on two dendrites. Middle, respective waveforms of bpAPs at different dendrite sites away from the soma. Right, magnification of the waveforms in the middle panels indicated by boxes. **C** Plot of the correlation between the amplitude ratio of bpAPs and the dendritic diameter ratio in dendrite pairs. Left, scatter plot of the relative (Rela.) amplitude ratio and diameter ratio; brown red, dendritic pairs with relative amplitude ratio ≥1, *n* = 99; grey, dendritic pairs with relative amplitude ratio <1, *n* = 59. Right panel, percentage of dendrite pairs showing a positive correlation between amplitude and dendritic diameter in groups with different diameter ratio ranges: total, 62%; diameter ratio >1.5, 82%; diameter ratio >2, 79%. **D** Plot of the correlation between the amplitude attenuation rate ratio of bpAPs and the dendritic diameter ratio in dendrite pairs. Left, scatterplot of the amplitude attenuation rate ratio (Amp. atten. ratio) and the diameter ratio; brown red, dendritic pairs with amplitude attenuation rate ratio ≥1, *n* = 89; grey, ratio <1, *n* = 69. Right, percentage of dendrite pairs showing a positive correlation between amplitude attenuation and dendritic diameter in groups with different diameter ratio ranges: total, 56%; when the diameter ratio >1.5, 63%; ratio >2, 67%. **E** Plot of the correlation between the amplitude attenuation rate ratio (Amp. atten. ratio) of bpAPs and the relative amplitude ratio in dendrite pairs. Rela. amplitude ratio, relative amplitude ratio. Brown red, dendritic pairs with amplitude attenuation rate ratio ≥1, *n* = 107; grey, ratio <1, *n* = 52. **F** A schematic diagram of examples of attenuating (Atten) and non-attenuating (Non-atten) bpAPs and the percentage of dendrites showing attenuating or non-attenuating propagation of bpAPs. Left upper, an attenuating bpAP; left lower, a non-attenuating bpAP. Right, brown red, attenuating propagation, 75%; grey, non-attenuating propagation, 25%. **G** Dendritic diameters of attenuating (*n* = 57) and non-attenuating (*n* = 51) bpAPs. *P* = 0.3231, unpaired *t*-test. Error bars, SEM. **H** Velocity of attenuating (*n* = 57) and non-attenuating (*n* = 45) bpAPs. *P* < 0.0001, unpaired *t*-test. Error bars, SEM.

In addition to bpAP velocity, bpAP amplitude is an important parameter reflecting the propagation process. Using an all-optical electrophysiological system, we recorded the relative amplitudes of bpAPs along dendrites and measured bpAP attenuation. Here, the relative fluorescence change was used to indicate the relative bpAP amplitude. The relative initial and average bpAP amplitudes and bpAP attenuation varied largely on different dendrites of the same neuron (Fig. 4B). As bpAP velocity is positively correlated with dendritic diameter, we explored the relationship between bpAP relative amplitude and dendritic diameter. Therefore, we divided dendrites into dendrite pairs as described above and integrated their relative initial bpAP amplitude ratio with the corresponding diameter ratio, taking the thinner dendrite as a reference. We found that 62% of dendrite pairs showed a positive correlation between the relative initial amplitude ratio and the diameter ratio, and this positive correlation became more significant as the diameter ratio increased (Fig. 4C). This finding indicated that bpAPs on dendrites with relatively large diameters are likely to also have relatively high amplitudes.

Previous *in vivo* studies have shown that the bpAP amplitude attenuates as the distance from the soma increases, and our experiments revealed similar bpAP attenuation dynamics. We investigated the factors that may affect the attenuation of bpAP amplitude. We first assessed the correlation between the attenuation rate and dendritic diameter, which revealed that 56% of dendrite pairs showed a positive correlation between the attenuation rate ratio and the diameter ratio. This finding may indicate a weak correlation, although this proportion was larger in the sets of dendrite pairs with diameter ratios >1.5 or 2 (Fig. 4D). Next, we associated the bpAP attenuation rate with the relative amplitude, which revealed that ~68% of dendrite pairs showed a positive correlation between the attenuation ratio and the relative initial amplitude ratio (Fig. 4E). This result indicated that, although the initial bpAP amplitude might be affected by dendritic diameter, the amplitude attenuation rate was more directly positively correlated with the initial amplitude. When bpAPs propagated along different primary dendrites of the same neuron, their initial amplitudes and attenuation rates varied markedly; bpAPs on thicker dendrites tended to have a higher amplitude and to attenuate more rapidly, which would be expected to lead to bpAPs with not much different amplitude at different distal dendrites.

Interestingly, not all bpAPs attenuated on dendrites, or, alternatively, they did not attenuate on the proximal dendrite sections included in our field of view. In total, 25% of bpAPs did not attenuate on the proximal dendrite (Fig. 4F). Therefore, we next explored factors that may control bpAP attenuation. We found no significant difference in diameter between dendrites in which bpAPs did and did not attenuate (Fig. 4G). In addition, we found that the velocity of non-attenuating bpAPs was higher than that of attenuating bpAPs (Fig. 4H). This finding indicated that non-attenuating bpAPs are more robust, and their velocity is more likely to be fast. We hypothesized that one or several dendrites may be responsible for delivering a signal to a distal dendrite as soon as possible, so the bpAP on such primary dendrites would be expected to be strong and non-attenuating.

### bpAPs Decelerate Significantly After Passing Through a Branch Point

**Fig. 5:**
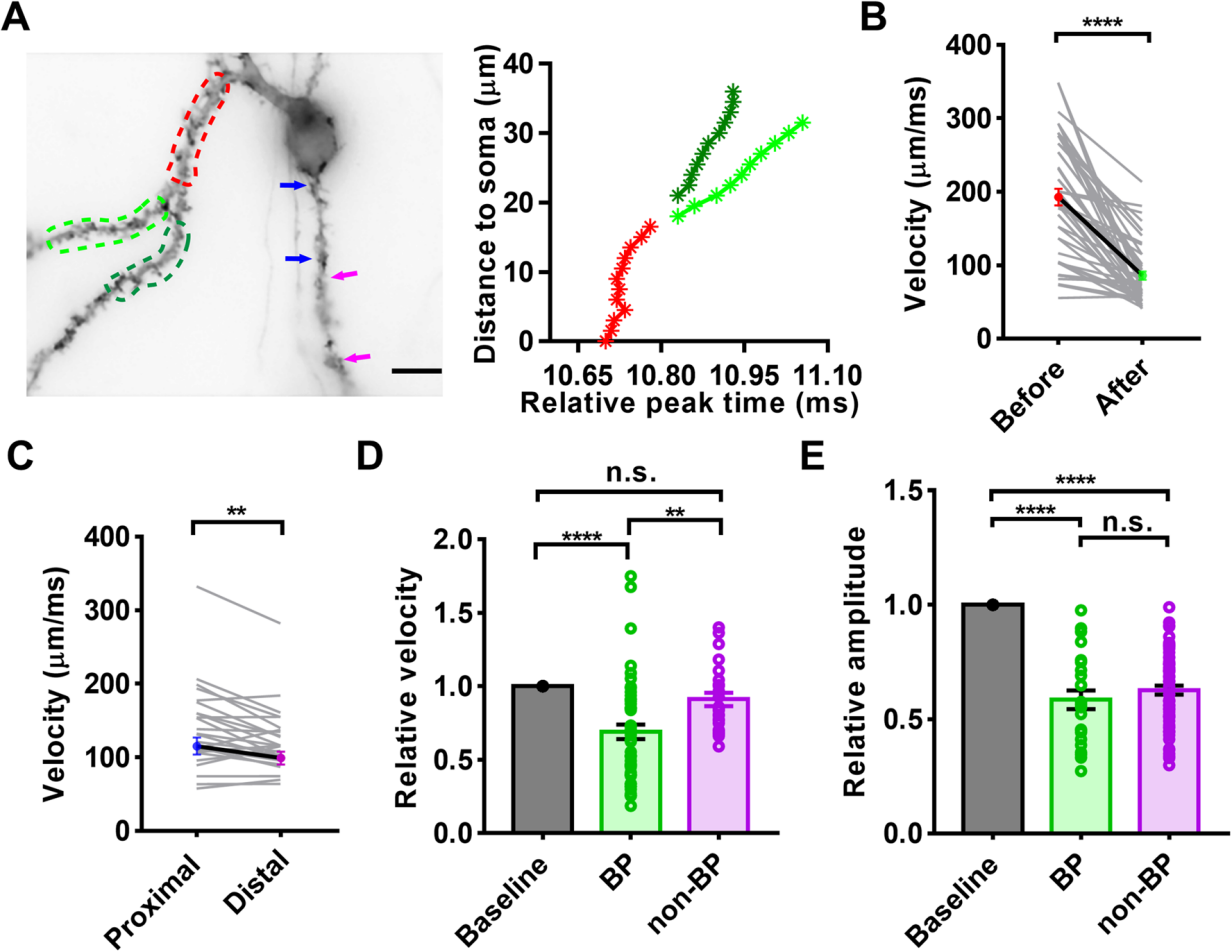
Branch points (BP) cause attenuation of bpAP velocity. **A** Left, a sample primary dendrite divided into two secondary dendrites. Right, peak time of bpAPs recorded at several points along the dendrite; the colors of lines represented the segments marked by dotted lines in the left panel (red, primary dendrite; light and dark green, secondary dendrite; blue arrows, proximal part; pink arrows, distal part. Scale bar, 10 µm. **B** bpAP velocity before and after a branch point. *n* = 50; *P* <0.0001, paired *t*-test. Error bars, SEM. **C** bpAP velocity at the proximal and distal parts of a long dendrite. *n* = 25; *P* = 0.0038, paired *t*-test. Error bars, SEM. **D** Relative bpAP velocity after passing through a branch point or propagating along a long dendrite. BP, branch point, *n* = 50; non-BP, non-branch point, *n* = 25; *P* <0.0001, *P* = 0.0034, *P* = 0.4747, respectively, one-way ANOVA. Error bars, SEM. **E** Relative amplitude of bpAPs after the branch point or propagation along a long dendrite. BP: *n* = 27, non-BP: *n* = 20, *P* <0.0001, *P* = 0.4900, *P* <0.0001, respectively, one-way ANOVA. Error bars, SEM.

Another important morphological feature of dendritic trees is their branching pattern. We found that bpAPs decelerate when they propagate from primary dendrites into secondary dendrites (Fig. 5A). Therefore, we performed experiments to determine how bpAPs change when they travel through a branch point.

First, we compared the velocities of bpAPs in dendrites before and after branch points. Most bpAPs decelerated significantly after a branch point (Fig. 5B). According to previous studies, bpAP amplitude attenuates naturally along dendrites without branch points. To determine whether bpAP self-attenuation influences velocity, we selected long primary dendrites and divided them into proximal and distal parts according to the distance from the soma, with each part 15–20 μm in length (Fig. 5A). In contrast to the bpAPs traveling through branch points, most bpAPs showed only slight changes in velocity at the distal parts of dendrites lacking branch points (Fig. 5C). Although a significant difference was found between the bpAP velocity at each part, the results did not indicate a significant decrease. We also compared the relative change in velocity between these two cases, and this revealed that the decrease in velocity after passing a branch point was significantly greater than that which occurred along a long dendrite (Fig. 5D). These results demonstrated that branch points play a major role in the bpAP velocity decrease that occurs when bpAPs traveled through a dendritic tree. In contrast to the change in velocity, the relative change in bpAP amplitude after a branch point did not differ significantly from that which occurred on a long dendrite (Fig. 5E). This finding indicated that branch points are not primarily involved in the attenuation of bpAP amplitude.

### Simulations Reveal a Significant Correlation Between BpAP Velocity and Dendritic Morphology

We applied modeling to gain a better understanding of how the dendritic morphology influences the velocity and amplitude of bpAPs. The passive parameters and gating kinetics of voltage-dependent Na^+^ and K^+^ channels used in the simulations referred to previous literature [26–30, 32]. First, we tested the influence of dendritic diameter on bpAP velocity. In the model, eight dendrites with diameters increasing from 0.5 μm to 4 μm incrementally were attached to the soma. In the model, the propagation velocity increased as the dendrites became thicker and the velocity of the bpAP was nearly linearly correlated with the square root of diameter ratio (data not shown).

**Fig. 6:**
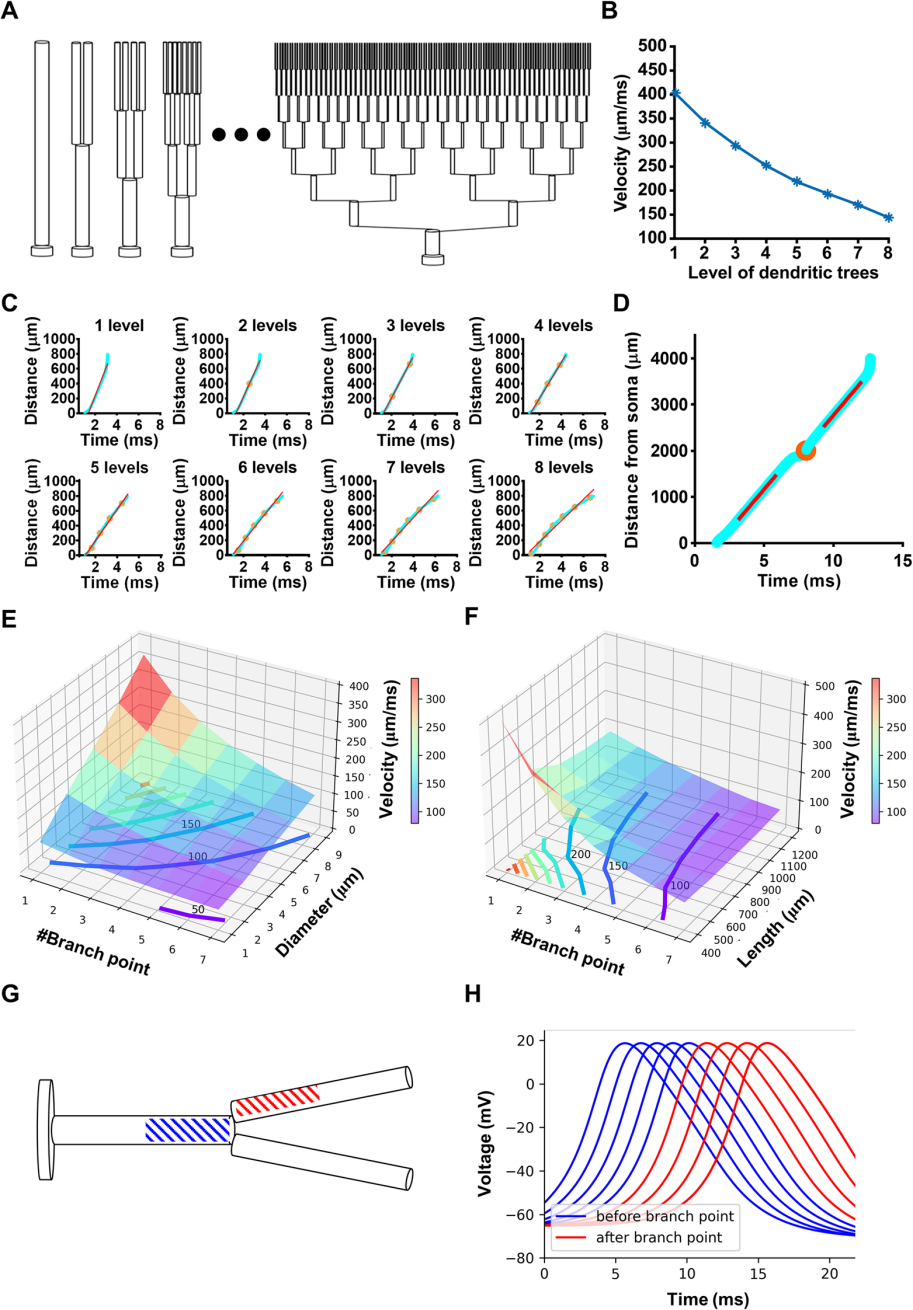
Simulations with different dendritic morphology. **A** Eight neuron models with increasing dendritic tree branch levels and the same total dendritic length (800 μm). **B** The average bpAP velocity on the eight dendritic trees of **A**. **C** The time of the bpAP peak from the soma to the end of the dendrite on the eight dendritic trees. Distance, distance to soma; the orange points mark branch points, and the red lines are linear fits of distance-time curves. **D** Same as **A**, but on one binary tree with one primary dendrite and two secondary dendrites. The three dendrites have the same diameter (5 μm). **E** The influence of the number of branch points and the equivalent diameters on the velocity of the bpAP. The step of contours is 50 μm/ms. **F** The influence of the number of branch points and the lengths of models on the velocity of bpAPs. The step of contours is 50 μm/ms. **G** A model with two levels of dendrites. The segments with blue and red slashes are located before and after the branch point. **H** The blue/red traces are voltages of blue/red segments in Fig. 5C. The branch point is located at 1000 μm. APs are recorded at 650, 750, 850, 950, 1150, 1250, and 1350 μm.

Then, we tested the influence of branch points on bpAP velocity. Eight neuron models with binary dendritic trees were constructed; these dendritic trees had the same total length, but their branching level increased incrementally, ranging from 1 to 8 levels (Fig. 6A). Here a “normal” situation was applied, in which the primary dendrite was 5 μm thick, and the diameters of two daughter dendrites after the branching point were mathematically equivalent to that of their mother dendrite (see Materials and Methods), so the dendritic diameter decreased as the dendritic level increased. All other passive and active parameters were the same in all dendrites. We recorded the AP propagation process, including passage through every branch point, and calculated the average velocity from the soma to the ends of the dendrites. The results showed that the average velocity along an entire dendrite decreased as the branching level of its dendritic tree increased (Fig. 6B); therefore, as the number of branch points passed by the bpAP increased, its velocity decreased (Fig. 6C). To simulate an extreme situation, we modeled a neuron with a binary dendritic tree, while the diameters of the primary dendrite and two secondary dendrites were unchanged. We found that the AP propagation velocity on the primary and secondary dendrites was nearly equivalent, except for a small change at the branch point (Fig. 6D). These results corresponded with our experimental findings under relatively conservative conditions. Taken together with the fact that the diameter of a dendrite always decreases after a branch point in actual neurons, this result led us to hypothesize that attenuation of bpAP velocity after a branch point might be the result of a reduction in dendritic diameter.

Next, we changed the number of branch points, dendritic diameter, and dendrite length of neuron models to examine how these parameters interact and regulate bpAP velocities. We first constructed a neuron model in which the total length of the dendritic tree was 800 μm. We adjusted its branch points from 1 to 7 or dendritic diameter from 1 μm to 9 μm (all levels of dendrites) and recorded the velocity of bpAPs. The results showed that the bpAP velocity was positively correlated with dendritic diameter when the number of branch points was fixed, and the bpAP velocity was negatively correlated with the number of branch points when the dendritic diameter was fixed (Fig. 6E). We then constructed a neuron model with a fixed dendritic diameter (5 μm) changeable total length and number of branch points. To sum up, the velocity of bpAP was negatively correlated with the density of branch points (Fig. 6F). The bpAP accelerated when it was near the end of the model, because the axial current was bigger. Therefore, in the model, the shorter the dendrite was, the velocity was influenced by the model boundary more and accelerated more.

Then, we tested how the number of branch points influences the attenuation of bpAPs. Here, we also used a neuron model with a binary dendritic tree and the diameters of the primary dendrite and two secondary dendrites were the same (5 μm) (Fig. 6G). We focused on the bpAP on the segments located on the segments before and after the branch points. As the active parameters were set uniformly along the whole dendritic tree, the AP shape (including the amplitude and half-width) was not changed during propagation (Fig. 6H). So under the conditions and parameters of our simulations, the branch point did not attenuate the bpAP.

## Discussion

The extent to which bpAPs invade distal dendrites is influenced in a complex manner by several factors, which include the pattern of excitatory and inhibitory inputs, ion channel distributions, and dendritic morphology. Here we focused on dendritic morphology, which has been considered to be the most decisive factor, to investigate how morphology specifically affects bpAPs. Using an all-optical electrophysiological method, we were able to record and study the propagation of bpAPs in proximal dendrites with relatively high spatial resolution in cultured neurons. By comparing bpAPs in the dendrites of one neuron, we found that the propagation velocity and amplitude of bpAPs were positively correlated with dendritic diameter. Another morphological parameter, the branching pattern, had a repressive effect on propagation. These results, taken together with the results from our computational neuron model, clarified how dendritic morphology affects bpAPs and indicated that dendrites perform distinct functions corresponding with their morphology, especially with regard to their participation in synaptic plasticity. We aimed to reveal a universal rule about the effect of dendritic morphology on bpAPs *in vitro*. And we established a complete process for recording and analyzing the propagation of bpAPs, which could be adapted to study the influence of other factors like ion channel distributions and synaptic inputs.

As a credible method of examining APs, voltage imaging has been used in many studies to measure their velocity and amplitude. However, in previous studies using dual-patch clamp methods, the average velocity along a section of dendrite (usually hundreds of micrometers in length) has been reported, and these data could not reveal the propagation process in detail, because dendrites vary along their lengths and have branch points. Voltage imaging provides a way to overcome such spatial restrictions; for example, bpAPs may be recorded in small dendritic regions such as spines [35]. By taking advantage of the fast response speed of QuasAr2, we were able to measure bpAP velocity in dendrite segments <20 μm in length. On the other hand, due to restrictions associated with technical limitations, it is difficult to record APs on several dendrites using electrophysiological methods, but voltage imaging enabled us to record APs in the soma and several dendrites at the same time at the micrometer scale. Therefore, we were able to study the entire process of AP propagation on a dendrite, and we compared differences in bpAPs before and after branch points, as well as on two homologous secondary dendrites. In addition, our method provided a way to link AP propagation with morphological parameters and quantitatively analyze their effects. The bpAP velocity recorded *via* patch-clamp was not exactly the same as that reported in previous studies, in which it was generally less than 1 mm/ms. This velocity was slightly faster than that reported in our study, and this change might have been the result of two different factors. First, the brain slices used in electrophysiological experiments provided neurons that more closely resembled cells under normal physiological conditions; such neurons were encircled and supported by glia and other brain tissue. Second, the experimental temperature for brain slice patch-clamp recordings was >30 °C in previous studies, whereas our recordings were performed at room temperature (23–25 °C). Several studies have found that the experimental temperature has a large influence on bpAPs (~34 °C in [36]; room temperature in [37]).

Theoretical studies have found that AP propagation velocity is directly proportional to neurite diameter [33, 34, 38, 39]. Our research focused on homologous primary dendrites and homologous secondary dendrites as a way of studying the influence of dendritic diameter. This strategy excluded other systematic differences such as different cellular states and fluorescent protein expression. We found that, in a large proportion of primary dendrites, bpAP velocity was positively correlated with dendritic diameter; this proportion was larger among secondary dendrites, indicating that secondary dendrites branching out from the same primary dendrite are more homogeneous in other characteristics. In addition, we were able to shorten the dendritic segments to obtain a more detailed picture of the variation in velocity along a long dendrite. Although this analysis could be improved, the results indicated that bpAPs do not propagate with a steady velocity along dendrites. The underlying mechanisms and functions of this fluctuation remain to be investigated in future studies. The branching pattern is another important morphological feature of dendrites. Previous studies recorded bpAPs between proximal dendrites and distal dendrites after several branching points, making it difficult to discern the influence of each branch point. Using voltage imaging, we recorded the bpAP propagation process in dendrite segments before and after branch points. As expected, the bpAP velocity decreased significantly after each branch point. In addition, further study of long dendrites indicated that self-attenuation has little effect on bpAP velocity.

Previous studies have shown that the velocities of bpAPs are very different not only in different types of neuron, but also in different dendrites of the same neuron, such as the apical and basal dendrites of pyramidal neurons. In our study, neurons were divided into pyramidal neurons and granulosa cells, the major excitatory neurons in the hippocampus. The results showed that the difference in velocities of bpAPs on the dendrites between these two types of neuron was not as large as in previous studies. We thought that this difference might be caused by the *in vitro* conditions. The growth environment plays a central role in the determination of neuronal dendritic morphology. Previous studies have shown that when neurons are isolated from the *in vivo* environment and induced to regenerate in culture dishes, certain specific dendritic morphology of neurons can be preserved, such as Purkinje cells from the cerebellum [40]. But this only happens in neurons with extremely specialized dendritic morphology. Many features of neuronal type-specific complex dendritic morphology depend on local neural signals and specific interactions with matrices and other cells. The dendritic trees of hippocampal pyramidal neurons cultured *in vitro* can become complex and multipole [41, 42], without differentiation into primary (apical) and secondary (basal) dendrites. Therefore, in this study, we suggest that *in vitro* culturing conditions may weaken some of the characteristics of pyramidal neuron dendritic trees and reduce some of the morphological differences in dendritic trees between pyramidal neurons and granule cells, making their morphology tend to become similar. The less significant difference in the bpAP between these two kinds of neuron may demonstrate that the varieties in dendritic morphology largely determine the differences in the properties and propagation of bpAPs in different neurons.

In addition to velocity, the peak amplitude is also an important parameter that partly reflects bpAP strength. Although it was difficult to measure the absolute value of bpAP amplitude using voltage imaging, we were able to compare the relative amplitude in the same neuron to study the effect of dendritic morphology. As with velocity, in most neurons, bpAP amplitude was also positively correlated with dendritic diameter. The bpAP amplitude on primary dendrites of the same neuron varied largely because of morphological and physiological variation. Similar to the results from previous studies, we found that most bpAPs attenuated significantly along dendrites, but ~25% of bpAPs showed nearly unattenuated propagation. In bpAPs attenuating in dendrites, the attenuation rate was also positively correlated with dendritic diameter in most neurons. This finding indicated that the amplitude of a bpAP on a thick dendrite is likely to be high, but it is also likely to attenuate quickly, so the amplitudes of different bpAPs at distal dendrites would be similar. The presence of these two types of bpAP, attenuated and unattenuated, may imply that several kinds of dendrite with distinct properties and functions are present in the brain.

Consistent with our experimental results, in neuron model simulations, the velocity of bpAPs in dendrites increased as the dendritic diameter increased. The velocity ratio and square root of diameter ratio of every dendrite pair showed a linear positive correlation, but the slopes of these correlations in the experimental results varied widely, and this variation did not occur in our simulation. This finding indicated that, under experimental conditions, the dendritic diameter has a large effect on bpAP velocity. Simulations in neuron models with multi-level dendritic trees also demonstrated that the velocity of bpAPs decreased after passing through a branch point when the dendritic diameter decreased after the branch point. However, the bpAP velocity remained almost unchanged when the dendritic diameter was identical before and after the branch point. Combined with the fact that most dendrites became thinner after the branch point, the simulation results indicated that the reduction in velocity after a branch point was mainly due to the decrease in dendritic diameter. The capability of branch points to physically stop propagation remains to be studied. In our simulations with an active propagation neuron model, the amplitude of bpAPs did not attenuate on long unbranched dendrites if the ion channel parameters were constant along the entire dendritic tree. This kind of propagation could be considered as propagation under perfect conditions. Therefore, attenuation of bpAP amplitude was mainly caused by changes in the type and density of ion channels. Combined with our results demonstrating a correlation between dendritic diameter and velocity, these findings allow general predictions to be made regarding the process of bpAP propagation.

In order to study the bpAP in an individual neuron, we used synaptic blockers to block synaptic inputs. But synaptic input also plays a modulatory role in affecting the propagation of bpAPs. The interaction of synaptic inputs and bpAP has been studied both in excitatory and inhibitory synapses. Timely excitatory inputs at the distal dendrites can enhance the bpAP and possibly trigger dendritic spiking and burst firing to support propagation of the active bpAP into more distal dendritic trees [12, 43–46]. In contrast, inhibitory inputs can attenuate the propagation of bpAPs [47–49]. Inhibitory signals through GABA receptors in a single spine attenuates bpAP-induced Ca^2+^ influx within the same spine [50]. And the effects of inhibitory inputs on the propagation of bpAPs are likely to be most influential in proximal regions of the dendritic tree in which the bpAP has not attenuated much. Studies of how the AP and the excitatory postsynaptic potential (EPSP) interact initially focused on Ia-type transient K^+^ current [51, 52]. The Ia-type transient K^+^ current attenuates bpAPs in hippocampal CA1 neurons, and Ia-type K^+^ channels that produce this current are deactivated by depolarizing synaptic input signals [53]. This provides an explanation for the mechanism of how timely EPSPs strengthen bpAPs. In general, the complex balance between multiple opposing synaptic signals determines the integration of synaptic input and bpAPs. The effect of synaptic inputs makes the determination of the extent of the bpAP more complex. So, we blocked synaptic inputs to focus on the effect of dendritic morphology on bpAP. Indeed, it is important and necessary to study the modulation of bpAPs by synaptic inputs in a more detailed way. By using the all-optical electrophysiological method, it will be convenient to compare bpAPs on the same dendrite with or without one or several kinds of synaptic signal and figure out the quantitative effects of synaptic inputs.

The induction of STDP requires the precise temporal order of presynaptic and postsynaptic depolarization in time windows of tens of milliseconds, and the bpAP might be an important source of postsynaptic depolarization. Although AMPA receptor activation is obligatory for STDP induction, the activation of NMDA receptors and bpAPs are necessary as well [54]. The STDP time window can be modulated by interactions between the EPSP and the bpAP [55]. An EPSP can strengthen subsequent bpAPs arriving within tens of milliseconds [12, 56]. And this boosting of bpAP can in turn modulate the magnitude of spike-timing-dependent LTP [11, 12, 57]. The velocity and amplitude of a bpAP directly determined the timing and strength when it reaches the synapse. Considering the effect of dendritic morphology on the velocity of the bpAP, we speculated that among the dendrites of each neuron, one or several dendrites may be primarily responsible for the transmission of neuronal information to distant synapses and the induction of synaptic plasticity. The interactions of EPSPs and bpAPs are more evident on a thick and less-branching dendrite in which the bpAP propagates faster and at a higher amplitude. This functional specialization of different dendrites also allows neurons to integrate and transmit neuronal signals more efficiently, which is crucial for the proper functioning of neuronal circuits.

## Supplementary Information

Below is the link to the electronic supplementary material.

Supplementary file1 (PDF 825 kb)

Supplementary file2 (MP4 100 kb)
