# Supplementary material for: Dendritic Morphology Affects the Velocity and Amplitude of Back-propagating Action Potentials
Source: Neurosci Bull. 2022 Aug 19;38(11):1330–46. doi: 10.1007/s12264-022-00931-9 (PMC9672184; doi:10.1007/s12264-022-00931-9)
Supplement: Supplementary file 1 — Supplementary file1 (PDF 825 kb) [file 12264_2022_931_MOESM1_ESM.pdf]

Supplementary Materials

Supplementary Figures

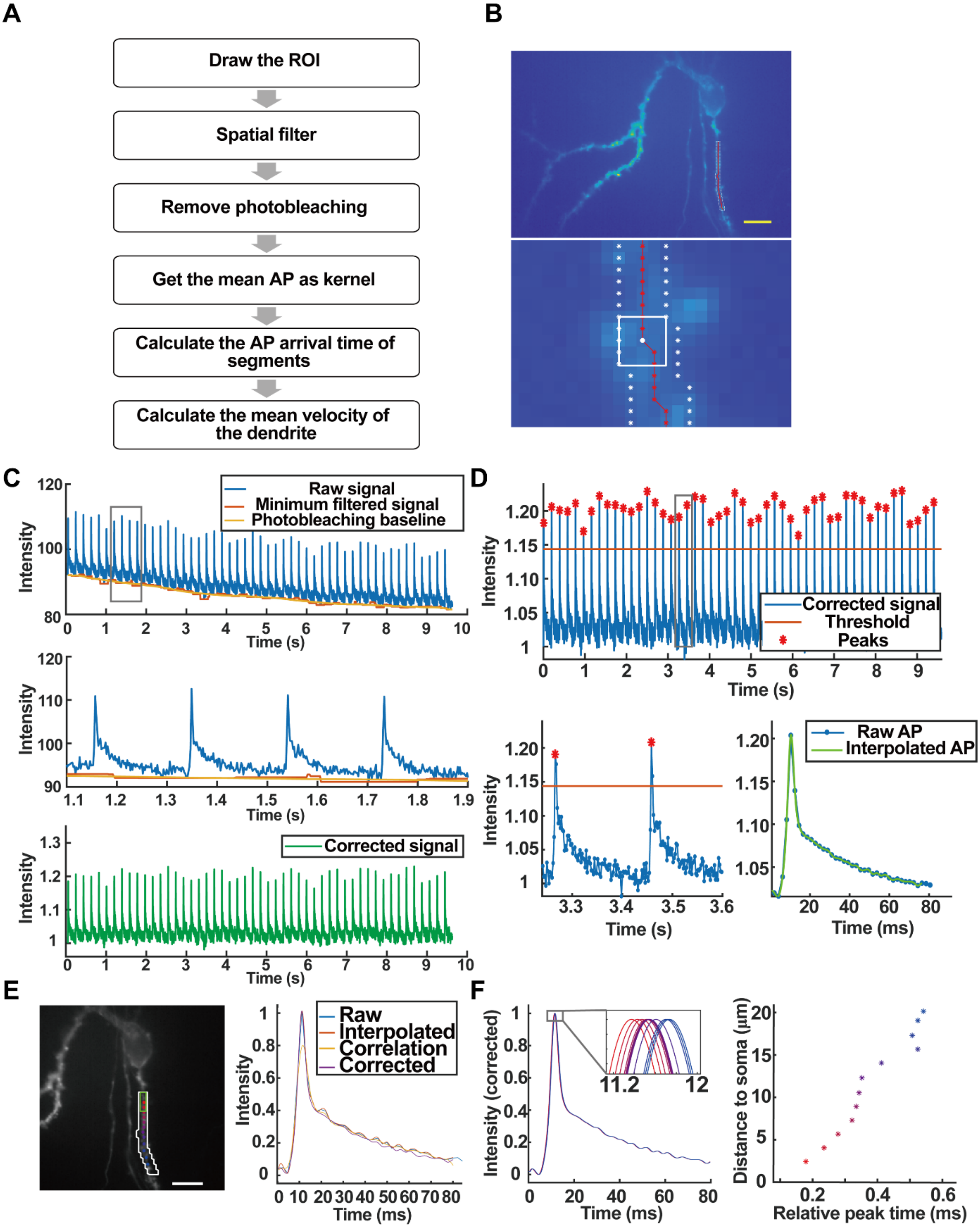

Fig. S1 Calculation of propagation velocity from raw data. **A** Flow chart of the data processing method. **B** Sample dendrite used for data processing. The red trace is the centerline of a dendrite drawn by the user; the white box (5 pixels × 5 pixels) in the lower panel is the region of interest (ROI). Scale bar, 10 μm. **C** Removing the effect of photobleaching. Upper, the blue trace is the original (raw) signal, the red trace is the minimum filtering result of the original signal, and the yellow trace is the photobleaching baseline, which was calculated by the mean filtering of the red trace. Middle, magnification of the signal marked by a gray box in the upper panel. Lower, the corrected signal of the ROI ($F_{\mathrm{ROI}}/F_{\mathrm{baseline}}$), which was calculated by dividing the original signal (blue) by the photobleaching baseline (yellow). **D** Mean AP calculation. Upper, peak detection of each AP; the blue trace is the corrected signal after removing the photobleaching baseline, and the red trace is a manual threshold. APs whose maximum voltage was higher than the threshold were considered normal and used to calculate the mean AP. Red stars are peaks of each AP. Lower left, magnification of the signal marked by a gray box in the upper panel. Lower right, the blue trace is the mean AP, i.e. mean of all selected APs aligned with AP peaks, and the green trace is the mean AP waveform after interpolation. **E** Correcting signals and calculating the peak time of APs on dendrites. Left, the region marked by the white line is the ROI; the region in the green box is the 11-pixel-long window. The central points, whose color gradually changes from red (close to the soma) to blue (far from the soma), are the final pixels used for the calculation of AP propagation. Right, the yellow trace is the correlation between the blue AP trace and the red AP trace; the purple trace is the corrected signal, which is the final result of this step. **F** Time-aligned corrected APs from different locations on a dendrite. Left, APs on different pixels (same as the central points in **E**) of the dendrite. Right, time of the bpAP peak and corresponding distance to the soma.

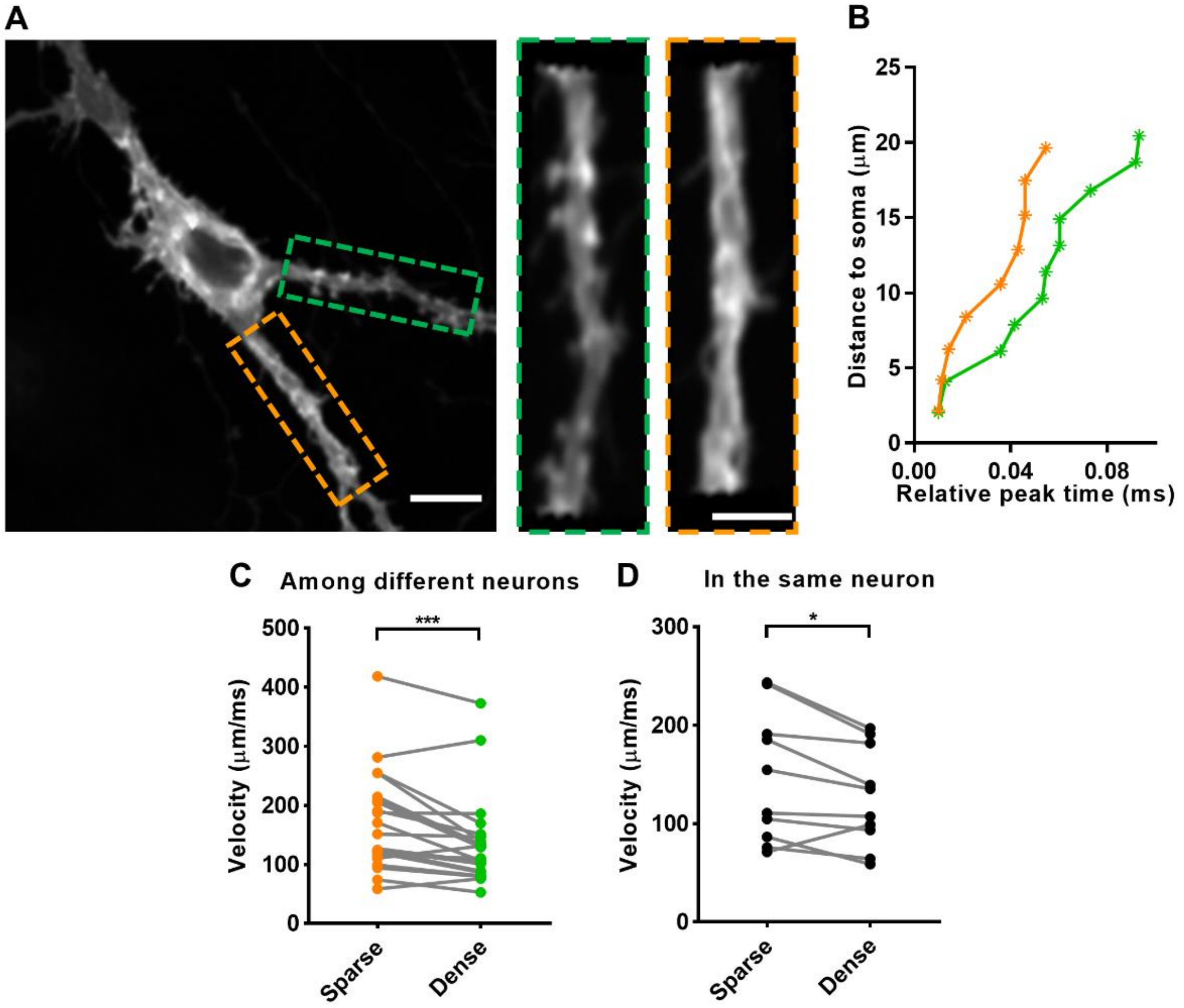

Fig. S2 The velocity of bpAPs is negatively correlated with spine density in dendrites with a similar diameter. **A** Representative fluorescence image of a neuron where two dendrites have different spine densities. Right, enlarged images of two dendrites marked by dashed lines with different colors in the left panel. Scale bar: left, 10 μm; right, 5μm. **B** Plot of the relative peak time of bpAPs on the two dendrites *versus* the distance to the soma. The color of the points and lines correspond with the dendrite in **A**. **C** Comparison of the velocity of bpAPs on the two groups of dendrites with different spine densities from different neurons. n = 21; P = 0.0008, paired t-test. **D** Comparison of the velocity of bpAPs in two groups of dendrites with different spine densities from the same neurons. n = 10; P = 0.0282, paired t-test.
